# Reframing Rehabilitation Retention in Long-Term Conditions: A Theory-Informed Mini-Review of Psychological Adaptation and Engagement

**DOI:** 10.3390/healthcare14172697

**Published:** 2026-08-24

**Authors:** Mohammad Khoshraftar, Zahra Karimi, Lyudmila S. Yermukhanova, Nurbakyt Ardak, Alireza Afshar

**Affiliations:** 1 Student Research Committee, Bushehr University of Medical Sciences, Bushehr 7518759577, Iran; khoshraftarm.mk@gmail.com; 2 RN Ross and Carol Nese College of Nursing, The Pennsylvania State University, State College, PA 16802, USA; zahrakarimi1532@gmail.com; 3 Department of Public Health and Healthcare with the Course of Nursing, Marat Ospanov West Kazakhstan Medical University, Aktobe 030019, Kazakhstan; yermukhanova@zkmu.kz; 4 Department of Public Health, Asfendiyarov National Medical University, Almaty 050012, Kazakhstan

**Keywords:** rehabilitation, chronic disease, treatment adherence and compliance, adaptation, psychological, patient dropouts

## Abstract

**Highlights:**

**What are the main findings?**

- Rehabilitation retention is organized into six phases: referral and eligibility, uptake or initiation, early attendance, ongoing attendance or dose, completion or planned discharge, and post-program maintenance.
- Patient disengagement may reflect interacting clinical, psychological, relational, organizational, and policy determinants, and several proposed pathways remain hypotheses.

**What are the implications of the main findings?**

- Rehabilitation programs should distinguish service access, stage-specific participation, time to dropout, planned discontinuation, clinical response, and longer-term maintenance.
- Targeting modifiable mechanisms may help reduce avoidable disengagement when rehabilitation remains safe, appropriate, beneficial, and consistent with patient goals.

**Abstract:**

Rehabilitation in long-term conditions requires not only clinical capacity but also sustained patient engagement, yet retention is commonly reduced to crude completion metrics. This theory-informed conceptual mini-review used a purposive, non-systematic synthesis of literature identified from the authors’ existing knowledge base, targeted PubMed/MEDLINE searches last updated on 9 August 2026, and backward reference checking; the final article cites 76 sources. Retention is reframed as six related phases comprising referral and eligibility, uptake or initiation, early attendance, ongoing attendance or dose, completion or planned discharge, and post-program maintenance, while time to dropout is treated as a longitudinal metric. Across these phases, disengagement may reflect interactions among clinical severity, symptom interpretation, illness beliefs, distress, motivational climate, therapeutic relationships, social context, and structural access. Four author-generated hypotheses concern psychological flexibility and acceptance, illness coherence and rehabilitation self-efficacy, autonomy-supportive care and therapeutic alliance, and social support and peer belonging. Direct evidence is concentrated in selected rehabilitation settings, whereas several stage-specific and cross-condition pathways remain theoretical extrapolations. Rehabilitation resilience is therefore proposed as a provisional interaction between patient adaptive resources and responsive care systems, not as a validated trait or a synonym for observable retention. Structural barriers may prevent participation directly, and discontinuation may be appropriate when treatment is unsafe, ineffective, burdensome, or inconsistent with informed patient goals. The framework is intended to guide prospective, condition-specific testing rather than imply causal validation or scientific consensus.

## 1. Introduction

Rehabilitation has become a central component of modern health care because the global burden of chronic disease, disability, multimorbidity, and functional limitation now requires health systems to move beyond survival and symptom control toward sustained participation, independence, and quality of life [1,2]. In long-term conditions, rehabilitation is not simply a technical intervention delivered by professionals but a structured process through which patients learn to live with symptoms, restore confidence, build self-management capacity, and translate clinical advice into everyday routines [1,3]. This process is behaviorally demanding because patients are often asked to attend repeated sessions, tolerate discomfort, accept delayed benefit, practice new skills, and maintain exercise or self-care habits after the protective structure of supervised care has ended [4,5]. Although rehabilitation programs can improve functioning and psychological well-being, many patients are not referred, never initiate treatment, attend irregularly, withdraw before completion, or fail to maintain therapeutic behaviors after discharge [6,7,8,9,10]. These patterns are commonly described with terms such as adherence, attendance, participation, completion, persistence, and maintenance, but the literature has not always distinguished these behaviors clearly enough [11,12]. This conceptual imprecision matters because a patient who does not attend the first session may face different barriers from a patient who begins enthusiastically and then withdraws when symptoms flare, confidence declines, or competing life demands accumulate [13]. For that reason, retention should be treated as a staged rehabilitation outcome that includes referral and eligibility, uptake or initiation, early attendance, ongoing attendance or dose, completion or planned discharge, and longer-term maintenance of activity or self-management [7,11,12,13,14,15]. This staged framing is especially important in cardiac and pulmonary rehabilitation, where evidence shows that referral, uptake, attendance, and completion are affected by a mixture of clinical severity, transport, cost, work demands, family responsibilities, illness beliefs, perceived benefit, psychological distress, and care context [6,8]. The same framing is also relevant to musculoskeletal, pain, neurological, and multimorbidity rehabilitation because patients in these settings often confront uncertainty, fear of movement, fluctuating symptoms, and difficulty integrating prescribed exercises into daily life [9,10]. A central limitation in the current literature is that dropout is often handled as an administrative failure or service-utilization endpoint rather than examined as a potentially heterogeneous event with clinical, psychological, relational, and structural explanations [16,17,18,19,20,21].

The unanswered question is not whether retention has many correlates, but how clinical, psychological, relational, organizational, and policy determinants can be organized across distinct phases without treating structural inaccessibility as deficient patient motivation [6,9,10,18,19,22,23]. Evidence also arises from populations with different symptoms, safety requirements, and service pathways, so cross-condition transfer should be treated as hypothesis generation rather than proof of common mechanisms [24,25,26,27]. In this review, interpreting avoidable dropout as a possible behavioral expression of adaptation is therefore an author-generated conceptual hypothesis rather than an established finding [4,16,17].

Psychological adaptation to chronic illness is not merely the absence of depression or anxiety, because it includes sense-making, acceptance, perceived control, self-efficacy, coping flexibility, social support, and the capacity to continue valued behavior under uncertainty [4,28]. These same constructs are plausible determinants of rehabilitation retention because patients must repeatedly decide whether the program is meaningful, safe, manageable, personally relevant, and worth continuing when benefit is gradual or symptoms are uncomfortable [29,30]. Psychological flexibility and acceptance are particularly relevant because they describe the ability to remain open to difficult internal experiences while acting in accordance with valued goals, a process that has strong support in chronic pain and acceptance-based rehabilitation research [31,32].

Illness coherence and self-efficacy provide a second pathway because patients are more likely to persist when they understand their condition, believe rehabilitation has a clear purpose, and feel capable of performing required behaviors despite symptoms or uncertainty. Autonomy-supportive communication and therapeutic alliance provide a third pathway because patients are more likely to internalize rehabilitation goals when clinicians support autonomy, competence, relatedness, shared decision-making, and collaborative problem-solving [24,28,33,34]. Social support and peer belonging provide a fourth pathway because family members, caregivers, peers, and group-based rehabilitation climates can reduce practical barriers, buffer emotional distress, normalize symptoms, and strengthen accountability [35,36]. Retention refers here to observable participation, whereas rehabilitation resilience is used provisionally for the interaction between a person’s adaptive resources and a system’s capacity to provide safe, accessible, responsive, and goal-concordant care [19,20,21,37,38]. The aim of this review is therefore to integrate psychological adaptation theory with rehabilitation retention research, distinguish evidence-supported relationships from theoretical extrapolations, and propose four testable hypotheses across staged outcomes [4,6,39,40].

## 2. Methods

This article is presented as a theory-informed conceptual mini-review whose primary objective is to integrate selected evidence and generate testable propositions, rather than estimate pooled effects or comprehensively map the field [39,40]. The focused mini-review format was selected because the question spans heterogeneous rehabilitation populations and theoretical constructs and requires critical conceptual synthesis within a concise article [39,40]. The review is explicitly non-systematic and should not be interpreted as a systematic or scoping review [39,40].

Sources were identified purposively from the authors’ existing literature base, targeted non-systematic searches in PubMed/MEDLINE, and backward reference checking, with the last targeted search conducted on 9 August 2026 [39,40]. Search concepts combined rehabilitation and condition terms with referral, uptake, initiation, attendance, completion, dropout, persistence, maintenance, psychological flexibility, acceptance, illness perceptions, self-efficacy, uncertainty, autonomy support, therapeutic alliance, social support, and structural barriers [39,40]. The search was iterative rather than protocol-based, and no fixed Boolean strategy, screening denominator, or duplicate selection process was prospectively recorded [39,40]. The synthesis was limited to English-language sources [39,40].

Priority was given to rehabilitation-specific primary studies and reviews involving adults with long-term physical conditions, together with measurement, theory, and methods papers that directly informed a retention phase or proposed mechanism [5,6,7,8,9,10,11,12,13,16,17,22,23,24,28,29,30,31,32,33,34,35,36,37,38,39,40,41,42,43,44,45,46,47,48,49,50,51,52,53]. Sources focused on acute care, medication taking, or non-rehabilitation outcomes were used only when their conceptual contribution was transferable to the review question [14,15]. The final manuscript cites 76 sources, including primary observational, qualitative, and intervention studies, reviews, measurement papers, theory and methods articles, and authoritative clinical guidance [27,39,40]. Evidence was interpreted narratively according to study design, directness to a rehabilitation retention outcome, consistency, and theoretical relevance, while conflicting findings were retained as evidence of heterogeneity [6,8,9,10,39,43,44]. No formal study-level risk-of-bias assessment or certainty grade was applied, which limits the strength of the synthesis [39,40]. The four mechanism families were retained because they had an explicit theoretical basis, available measures, potential modifiability, and plausible relevance to at least one retention phase [24,29,30,31,32,33,34,35,36,37,46,47,48,49,50,51,52,53].

## 3. Retention as a Staged Rehabilitation Outcome

A useful framework for rehabilitation retention should begin by refusing to collapse all participation behavior into a single completion variable, because engagement is better described as a sequence of linked but distinct behaviors. Although adherence terminology was originally clarified in medication research through the distinction between initiation, implementation, and persistence, the same conceptual logic applies to rehabilitation because patients must first start, then repeatedly attend, then complete, and finally maintain behavior after structured treatment has ended [11,12,13,14,41]. This distinction is clinically important because a patient who never starts rehabilitation may need referral endorsement, health-literacy support, transport solutions, and clearer orientation, whereas a patient who drops out after several sessions may need help with fear, fatigue, symptom interpretation, mood, or confidence [5,42]. It also matters for research because different predictors may operate at different phases, and a single crude completion outcome can hide whether a mechanism is associated with initial uptake, dose received, early dropout, or longer-term behavioral maintenance [7,9,11].

In cardiac rehabilitation, prospective studies and registry analyses have shown that non-participation and non-completion are shaped by demographic, clinical, psychosocial, and service-level factors, which supports the view that retention is multidimensional rather than purely logistical [6,8]. The qualitative cardiac rehabilitation literature also shows that patients’ decisions to attend are influenced by perceived need, physician endorsement, understanding of program value, social encouragement, work and family responsibilities, and fit between the program and everyday life. Pulmonary rehabilitation research similarly identifies environmental barriers, poor knowledge, beliefs about consequences, transport difficulties, symptom burden, and program characteristics as recurring determinants of referral, participation, and completion [5,9,10]. Studies of pulmonary rehabilitation completion further show that baseline clinical status, smoking, dyspnea, anxiety, depression, and social factors may influence dropout, although findings vary by setting and population [43,44]. These findings do not support a simple explanation in which patients leave rehabilitation because they are unmotivated or noncompliant; instead, they suggest that disengagement often emerges when program demands exceed the patient’s available psychological, social, and practical resources [45].

**Figure 1:**
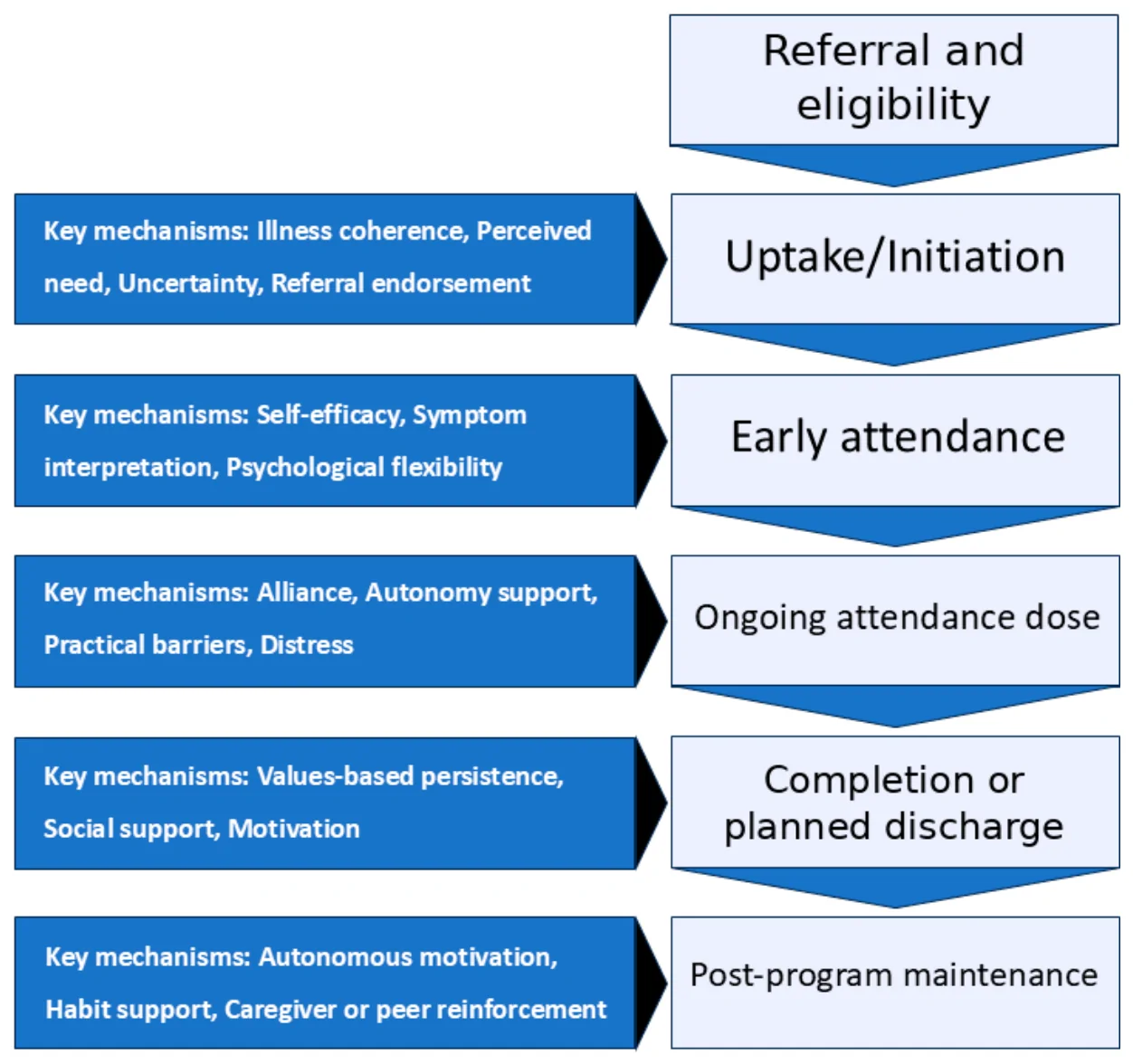
Author-proposed six-phase rehabilitation retention pathway. Time to dropout is a longitudinal metric during active treatment rather than a separate phase, and the stage-specific mechanisms remain hypotheses [7,11,12,13,14,15].

The exercise-adherence literature in rehabilitation and physiotherapy reinforces the same point by showing that adherence is influenced by self-efficacy, motivation, therapeutic alliance, beliefs about treatment, perceived barriers, symptoms, and contextual fit [22,23]. Measurement remains a major challenge because exercise-adherence studies frequently use different definitions, reporting methods, thresholds, and time frames, which makes it difficult to compare findings across conditions or interventions [12,41]. For the purposes of this review, retention should therefore be reported across six phases: referral and eligibility, uptake or initiation, early attendance, ongoing attendance or dose, completion or planned discharge, and post-program maintenance [7,11,12,13,14,15] (Table 1). Time to dropout is a longitudinal metric during active care rather than a separate phase [11,12,13]. These indicators are not equivalent to clinical outcomes because attendance and completion describe exposure to care, whereas symptoms, function, goal attainment, perceived benefit, adverse events, and treatment burden describe patient consequences [1,15,20,21]. The stage-specific mechanisms shown in Figure 1 remain hypotheses that require prospective evaluation across clinical populations [5,42,54].

Measurement is feasible only when the numerator, denominator, and timing are prespecified [12,13,23,41]. Studies should distinguish prescribed sessions, attended sessions, service cancellations, patient-missed sessions, dose modifications, treatment duration, and reasons for discontinuation [12,13,23,41]. Completion may reflect protocol dose, agreed goal attainment, or planned discharge, and maintenance should be assessed at explicit follow-up points, such as 3, 6, and 12 months, when feasible [5,13,15,21,42]. Administrative indicators of care exposure should be reported separately from symptoms, function, adverse events, perceived benefit, goal attainment, and treatment burden [1,15,20,21].

## 4. Psychological Adaptation Pathways

Psychological adaptation to long-term physical health conditions is a dynamic process through which patients make sense of illness, regulate emotional responses, maintain valued roles, develop coping strategies, and integrate health demands into daily life. Resilience in chronic disease is therefore better understood as adaptive capacity rather than as a fixed personality trait, and this framing is useful for rehabilitation because programs repeatedly expose patients to challenge, uncertainty, delayed reward, and temporary setbacks [4,55]. In this context, retention may be examined as an observable behavior potentially related to adaptation, but it is not evidence of resilience by itself [4,19,28]. Psychological flexibility and acceptance are especially relevant because they explain how patients may continue valued action even when pain, breathlessness, fatigue, fear, embarrassment, or discouragement cannot be fully removed before action begins [24,29].

For conceptual clarity, adherence refers to correspondence with agreed recommendations, while persistence refers to the duration of continued participation [11,12,13,14]. Engagement is broader and may include cognitive, emotional, and behavioral involvement, whereas maintenance refers here to continued exercise or self-management after supervised care [11,12,13,14,15]. Rehabilitation resilience is not treated as a validated trait or outcome but as a provisional multilevel interaction between patient adaptive resources and care-system responsiveness; retention remains the observable outcome [4,19,55]. Until a distinct measure and discriminant validity are established, studies should assess the proposed component constructs separately [30,34,50,53,56,57].

One important adaptation pathway involves the interpretation of symptoms as either manageable signals within rehabilitation or threatening signals that require avoidance. In chronic pain research, acceptance and psychological flexibility are associated with better functioning and can mediate improvement in acceptance-based treatments, which supports their relevance to rehabilitation contexts where symptoms are expected to fluctuate [25,58,59]. Psychological flexibility may support retention by reducing experiential avoidance, weakening catastrophic interpretations, and allowing patients to remain connected to personally meaningful goals even when immediate comfort declines. This pathway can be extended cautiously across long-term conditions because pain, dyspnea, fatigue, and emotional distress differ biologically but often share a behavioral consequence: the patient may withdraw from activities that are perceived as threatening, unrewarding, or beyond personal control [24,33,60,61].

Cross-condition extension requires clear clinical boundaries because symptom meanings and safety implications differ substantially [6,9,24,25,26,27]. Pain during graded musculoskeletal activity is not equivalent to chest pain, marked desaturation, acute neurological symptoms, or severe dyspnea [25,27]. Acceptance and flexible action should therefore be considered only after condition-specific assessment, red-flag evaluation, and individualized guidance and should never imply persistence through unsafe symptoms [1,25,27]. Clinical severity may directly affect eligibility, tolerability, dose modification, or discontinuation, while psychological and social factors may interact without necessarily mediating these effects [6,8,19,43,44,45].

A second adaptation pathway concerns illness coherence, perceived control, and self-efficacy. Patients are more likely to attend rehabilitation when they understand why it matters, believe the condition can be influenced by their own actions, and view symptoms during activity as interpretable rather than confusing or dangerous [30,32]. Self-efficacy theory further suggests that patients who believe they can execute required behaviors are more likely to initiate action, persist under difficulty, recover after setbacks, and translate guidance into practice. Condition-specific measures such as the Pulmonary Rehabilitation Adapted Index of Self-Efficacy are useful because confidence in rehabilitation is not identical to general optimism or generic coping confidence [31,34,62]. Intolerance of uncertainty may act as a mediator or amplifier because patients who experience uncertainty as highly threatening may interpret variable progress, temporary symptom provocation, or unclear timelines as evidence that rehabilitation is unsafe or ineffective [28,63].

Broader exercise-adherence research supports these adaptation pathways by repeatedly identifying self-efficacy, intention, planning, perceived barriers, treatment beliefs, and affective responses as correlates of adherence [22,23]. Rehabilitation studies also suggest that symptom severity alone is insufficient for predicting retention because psychological profiles, distress, beliefs, support, and contextual resources may influence whether patients remain engaged under similar clinical burdens [43,44]. A rehabilitation-resilience model should nevertheless recognize that clinical severity and structural access may directly determine eligibility, tolerability, dose modification, or discontinuation [4,6,18,19]. Symptom interpretation, uncertainty tolerance, confidence, and valued goals may explain selected patterns of staying or leaving, but they should not be assumed to mediate every clinical or structural barrier [4,6,19,20]. This interpretation does not imply that dropout is the patient’s fault; instead, it suggests that rehabilitation systems should support adaptation while also removing preventable access and service barriers [18,19,33,61].

## 5. Relational and Social Ecology of Retention

Psychological adaptation in rehabilitation is never purely intrapersonal because the motivational climate created by clinicians can either support or erode the patient’s willingness to persist. Self-Determination Theory suggests that patients are more likely to sustain behavior when the care environment supports autonomy, competence, and relatedness [35,46,47,48]. In cardiac rehabilitation, self-determined motivation predicts independent home-based exercise after program completion, and autonomy support from staff is linked to more self-determined motivation. This matters for long-term conditions because rehabilitation asks patients to internalize exercise, symptom management, and lifestyle change rather than merely comply with supervised instructions [35,47,49]. Measures such as the Health Care Climate Questionnaire make this pathway testable because they assess whether patients experience clinicians as autonomy-supportive rather than controlling [50].

Therapeutic alliance adds another relational layer because patients are more likely to disclose barriers, negotiate goals, and tolerate difficult treatment tasks when they feel understood, respected, and collaboratively guided. In physiotherapy and musculoskeletal rehabilitation, alliance has been associated with adherence, outcomes, and the patient’s ability to integrate exercise into daily life [16,36,51,52]. The availability of rehabilitation-adapted Working Alliance Inventory measures strengthens this field because alliance can be measured as an empirical mechanism rather than invoked as a vague clinical virtue [53]. Mechanistically, a strong alliance may help patients interpret pain, breathlessness, fatigue, or slow progress as manageable parts of therapeutic work rather than as signs of failure or abandonment by care providers [36,52]. This relational pathway may be particularly relevant during the early sessions, when patients are still assessing whether rehabilitation feels safe, relevant, respectful, and worth the effort [16,23]. Alliance is also dynamic and bidirectional because perceived benefit, symptom response, goal agreement, cultural fit, and clinician adaptation may alter the relationship over time [16,36,51,52,53]. A strong alliance should support continuation when appropriate but also shared modification or discontinuation when treatment is ineffective, burdensome, unsafe, or misaligned with patient goals [20,21,52].

**Figure 2:**
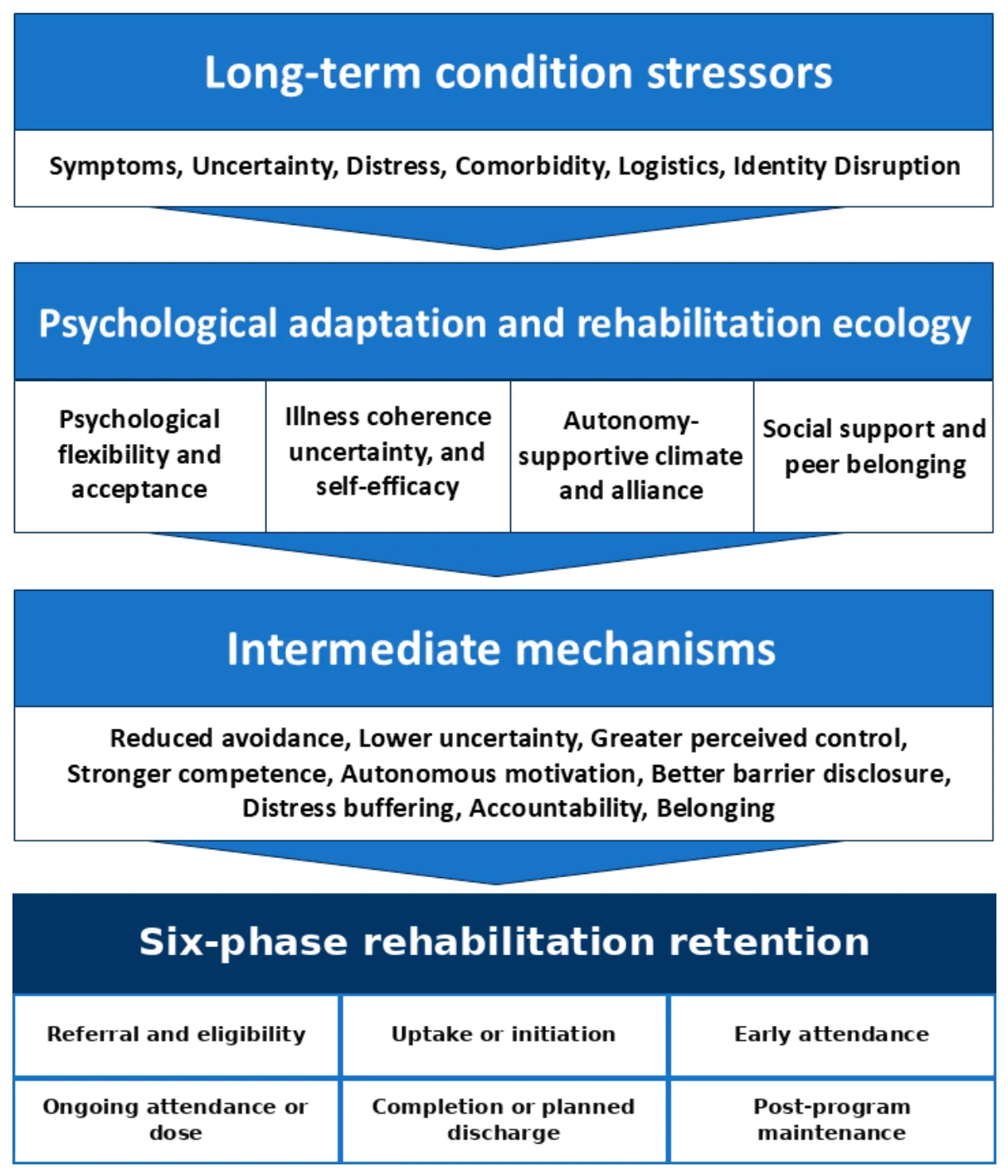
Author-proposed conceptual model of rehabilitation resilience. The arrows represent hypothetical pathways; reciprocal effects are possible, and the model has not been empirically validated [4,6,19,54].

Social support completes the ecology of retention because rehabilitation behavior occurs inside households, workplaces, communities, transport systems, and peer groups rather than inside clinics alone. In COPD, social support has been associated with self-care behaviors and pulmonary rehabilitation participation, suggesting that support can influence concrete treatment behavior rather than only emotional well-being [37]. Qualitative pulmonary rehabilitation research also shows that patients value belonging, shared experience, peer comparison, and supportive group relationships, which may strengthen confidence and reduce isolation [64,65]. Family-oriented pulmonary rehabilitation models further suggest that caregivers are not passive observers because they may support attendance, coping, symptom monitoring, and shared adaptation to chronic disease [18,38,64,66]. Cardiac rehabilitation research similarly identifies mood symptoms, social support, expectations, and perceived program fit as possible influences on adherence and continuation [10,67,68]. A reasonable synthesis is that support may operate through emotional buffering, practical help, accountability, identity change, and normalization of rehabilitation struggles, although identity-related mechanisms require more direct testing across conditions (Table 2) [64,67,68]. Together, these mechanisms form an author-proposed rehabilitation-resilience framework in which long-term condition stressors are hypothesized to relate to staged retention through psychological adaptation, relational care, and social support mechanisms (Figure 2) [4,6,19,37,54].

Social support is not uniformly beneficial, and its effects depend on source, function, timing, and fit with patient preferences [37,64,66,68,69]. Critical, controlling, conflictual, or overprotective responses may undermine autonomy and self-management rather than strengthen retention [69]. Support measures should therefore capture negative interactions and autonomy support instead of assuming that caregiver presence is protective [37,64,66,68,69].

**Figure 3:**
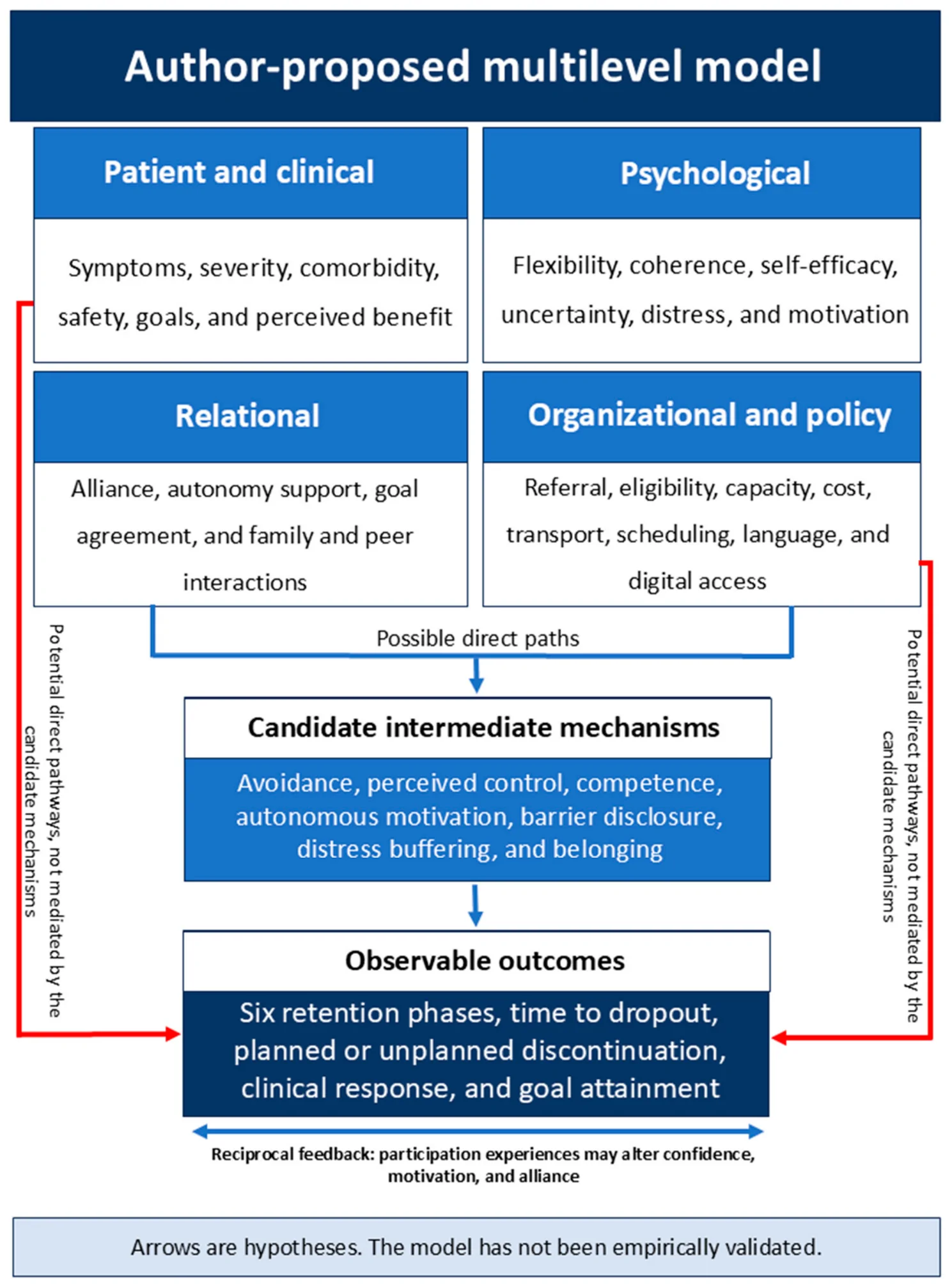
Author-proposed multilevel extension of the rehabilitation-resilience framework. Structural and clinical determinants may act directly, while participation experiences may alter confidence, motivation, and alliance over time. All arrows are hypotheses, and the model has not been empirically validated.

Psychological and relational mechanisms operate within organizational, community, and policy conditions that may affect participation directly [18,19,20]. Transport unavailability, unaffordable care, inflexible scheduling, limited capacity, language barriers, discrimination, and digital exclusion can prevent referral or attendance without psychological mediation [6,9,10,18,19,20]. Figure 3 therefore extends the mechanism-focused model by placing patient and clinical, psychological, relational, and organizational or policy determinants in parallel, with direct and reciprocal pathways treated as hypotheses [19,54].

## 6. Hypotheses and Mechanistic Pathways

The four pathways below are author-generated hypotheses derived from the preceding synthesis and should not be interpreted as established mechanisms [4,6,39,40] (Table 3). They combine condition-specific rehabilitation evidence with adjacent theoretical evidence, and Table 4 makes this distinction explicit [6,9,22,23,24,36]. Each hypothesis should be examined against more than one retention phase because the same mechanism may have different associations with initiation, attendance, completion, and maintenance [11,12]. Prospective studies should compare direct, mediated, moderated, and reciprocal models while establishing temporal ordering [47,54,61].

The proposed constructs are theoretically distinguishable but may be correlated, so entering all of them as parallel predictors may create multicollinearity and obscure temporal ordering [46,47,48,49,50,51,52,53,54]. Competing models should test whether autonomy support changes self-efficacy, alliance changes illness coherence, social support modifies distress, or early participation feeds back to confidence and motivation [47,48,52,54,68]. To limit participant burden, studies should prespecify one primary mechanism, one stage-specific outcome, clinical severity, and key structural confounders, with secondary constructs measured only when required by the causal model [12,23,41,54].

### 6.1. Psychological Flexibility and Acceptance

#### 6.1.1. Mechanistic Rationale

We hypothesize that greater psychological flexibility and illness acceptance at baseline, or early gains during treatment, may be associated with better attendance, completion, and maintenance, partly through reduced experiential avoidance and values-based action [24,25,29,58,59,60,61,70,73]. The rationale begins with the observation that many long-term conditions produce symptoms that cannot be fully removed before meaningful action becomes necessary [24,29,70]. When patients interpret pain, breathlessness, fatigue, palpitations, or weakness as signs of danger, they may reduce activity, miss sessions, and gradually disengage from rehabilitation [25,29]. Acceptance-based models propose that suffering and disability can be intensified when patients organize behavior around avoiding internal experiences rather than pursuing valued action [25,29,58,59]. In rehabilitation, this means that a patient may withdraw not only because symptoms are severe but because symptoms become fused with meanings such as danger, failure, shame, or loss of control [25,29,58,59,73].

Psychological flexibility can be understood as a resilience process because it allows patients to notice difficult symptoms and emotions without allowing them to completely dictate behavior [33,60]. Its components include acceptance, cognitive defusion, present-moment attention, values clarification, committed action, and flexible perspective-taking. Chronic pain reviews and mediation studies show that acceptance and psychological flexibility are associated with improvements in pain-related functioning, depression, anxiety, and disability in acceptance-based interventions [24,59,61]. These findings justify the hypothesis that early increases in flexibility may help patients remain in rehabilitation when initial sessions provoke discomfort, uncertainty, or disappointment [58,59]. The proposed bridge across conditions is not that pain and pulmonary or cardiac symptoms are identical but that avoidance, fear, low confidence, and values conflict can occur across many rehabilitation populations [17,33].

#### 6.1.2. Pathways to Staged Retention

The first pathway links flexibility to attendance through reduced symptom-triggered avoidance. Patients with higher flexibility may be more able to attend sessions despite discomfort because they can distinguish unpleasant sensations from catastrophic evidence that rehabilitation is unsafe [24,61,70]. The second pathway links flexibility to completion through values-based persistence because rehabilitation tasks become connected to personally meaningful goals such as walking with family, returning to work, preserving independence, or reducing reliance on acute care [33,59]. The third pathway links flexibility to maintenance because patients who practise committed action during supervised care may be more likely to continue exercise after discharge, when external structure and clinician contact decline [5,29,73].

This hypothesis can be operationalized with validated acceptance or flexibility instruments such as the Chronic Pain Acceptance Questionnaire, the CPAQ-8, the Psychological Inflexibility in Pain Scale, or broader psychological flexibility measures when the condition is not pain-specific [56]. Retention should be modeled with uptake, attendance proportion, completion, time to dropout, and follow-up maintenance, rather than only a binary end-of-program outcome [11,12]. The most informative design would assess flexibility at baseline and during the first few sessions to test whether early change predicts later completion above baseline distress, symptom severity, and logistical barriers [59,61]. Clinically, this hypothesis supports brief ACT-informed onboarding, symptom-normalization conversations, values-based goal setting, and early check-ins for patients who show high fear, avoidance, or ambivalence before nonattendance becomes established [24,26].

### 6.2. Illness Coherence, Uncertainty, and Rehabilitation Self-Efficacy

#### 6.2.1. Mechanistic Rationale

We hypothesize that higher illness coherence and rehabilitation self-efficacy at intake may be associated with better retention and that lower uncertainty and greater perceived control may mediate selected stage-specific associations [30,32,54,63,71]. This hypothesis is grounded in illness representation theory, which proposes that patients form beliefs about the identity, cause, timeline, consequences, controllability, and emotional meaning of illness [30,32,71]. When these beliefs are coherent and rehabilitation is perceived as relevant, patients are more likely to understand why repeated attendance is necessary even if improvement is slow [30]. In cardiac rehabilitation, illness perceptions have been linked to attendance, which suggests that patients’ interpretations of illness can influence concrete rehabilitation behavior [32,71]. This is clinically plausible because patients who do not understand the purpose of rehabilitation may interpret it as optional, unsafe, irrelevant, or less urgent than medication and acute care [9,10,32].

Self-efficacy adds a related but distinct mechanism because it refers to confidence in one’s ability to perform behaviors required to achieve desired outcomes [62]. In pulmonary rehabilitation, the PRAISE tool was developed to assess rehabilitation-specific self-efficacy, and higher self-efficacy has been linked with more favorable behavior after pulmonary rehabilitation [31,34]. General chronic disease self-efficacy measures may also be useful for cross-condition studies, although condition-specific tools can better capture confidence in breathing control, exercise tolerance, pacing, or symptom management [57,62]. Illness uncertainty may weaken both coherence and self-efficacy because patients who find uncertainty intolerable may interpret fluctuating symptoms, variable progress, or temporary setbacks as evidence that rehabilitation is ineffective or risky. This mechanism is especially relevant in long-term conditions because the trajectory is often unpredictable, and patients may need to act before they feel fully certain about prognosis, safety, or expected benefit [28,63].

#### 6.2.2. Pathways to Staged Retention

The first pathway links illness coherence to uptake because patients who understand the rationale for rehabilitation may be more likely to accept referral and attend the first session [32,71]. The second pathway links self-efficacy to early attendance because patients who believe they can perform rehabilitation tasks are more likely to return after initial difficulty [10,30]. The third pathway links uncertainty tolerance to completion because patients who can tolerate variable progress are less likely to interpret temporary symptom provocation or slow improvement as evidence that continuation is pointless [34]. The fourth pathway links perceived control to maintenance because patients who internalize rehabilitation as a manageable self-care strategy may be more likely to continue exercise or symptom-management practices after discharge [5,28,63].

This hypothesis can be tested using the Brief Illness Perception Questionnaire or related illness-perception measures, combined with self-efficacy tools such as the Self-Efficacy for Managing Chronic Disease scale or PRAISE in pulmonary populations. Uncertainty can be modeled as a mediator, moderator, or parallel predictor depending on the design and measurement schedule [28,30,63]. The strongest design would measure illness coherence and self-efficacy at intake, reassess confidence after early mastery experiences, and examine whether these changes predict attendance dose and formal completion. Clinically, this hypothesis supports individualized orientation, expectation setting, graduated task mastery, feedback on progress, and explicit discussion of normal symptom fluctuation during rehabilitation [4,31,34,57].

### 6.3. Autonomy-Supportive Climate and Therapeutic Alliance

#### 6.3.1. Mechanistic Rationale

We hypothesize that a more autonomy-supportive rehabilitation climate and stronger therapeutic alliance may be associated with attendance, completion, and maintenance through autonomous motivation, competence, and barrier disclosure [35,36,46,47,48,49,50,51,52,53]. Self-Determination Theory proposes that sustained behavior is more likely when the social environment supports autonomy, competence, and relatedness [47,49]. Cardiac rehabilitation research supports part of this pathway because self-determined motivation has been associated with independent home-based exercise, and autonomy support has been linked to more self-determined motivation for exercise [47,48]. This is important because rehabilitation requires more than short-term compliance; it requires patients to internalize the value of behavior and continue action when supervision, reminders, and external pressure decline [35,47,48]. Measures such as the Health Care Climate Questionnaire allow researchers to assess the patient’s perception of autonomy support rather than relying only on clinician self-report or program intention [50].

Therapeutic alliance strengthens this pathway by capturing agreement on goals, agreement on tasks, and the relational bond between patient and clinician. A strong alliance may promote retention because patients are more likely to discuss barriers, negotiate exercise modifications, accept realistic expectations, and continue after setbacks when they trust the clinician and feel respected [16,36,51,52]. Rehabilitation-adapted Working Alliance Inventory measures make this mechanism testable in rehabilitation settings. Alliance is not merely a pleasant interpersonal feature because it may shape the patient’s interpretation of effort, discomfort, feedback, and temporary failure [36,52,53]. This hypothesis therefore places part of psychological adaptation inside the care relationship rather than treating it only as an internal patient trait [16,23].

#### 6.3.2. Pathways to Staged Retention

The first pathway links autonomy support to uptake and early attendance by increasing the patient’s sense that rehabilitation participation is personally meaningful rather than externally imposed [35,47]. The second pathway links competence support to attendance dose because patients who receive constructive feedback, graded challenges, and realistic encouragement may feel more capable of continuing [46,49]. The third pathway links relatedness and alliance to completion because patients who feel understood and collaboratively guided may be less likely to disengage silently when barriers emerge [47,48]. The fourth pathway links autonomous motivation to maintenance because internalized motivation is more likely than controlled motivation to survive after formal supervision ends [36,52].

This hypothesis can be operationalized with perceived autonomy-support scales, Working Alliance Inventory variants, need-satisfaction measures, and behavioral-regulation scales [50,53]. Cluster randomized trials would be particularly useful because rehabilitation teams, rather than individual patients, can be trained in autonomy-supportive communication, shared goal setting, reflective listening, and collaborative problem-solving [36,52]. Repeated early measurement is important because alliance and motivation may develop or deteriorate rapidly during the first sessions, which may also be the decisive window for dropout risk [35,47]. Clinically, this hypothesis supports staff training in noncontrolling language, shared decision-making, personalized goal work, expectation management, and early barrier disclosure [6,52].

### 6.4. Social Support and Peer Belonging

#### 6.4.1. Mechanistic Rationale

We hypothesize that appropriately matched social support and peer belonging may moderate the association between distress and rehabilitation retention, with potentially larger benefits among patients with higher anxiety or depressive symptom burdens [37,44,54,67,68]. Psychological distress can interfere with rehabilitation by reducing energy, increasing avoidance, amplifying threat perception, weakening concentration, and making ordinary practical barriers feel less manageable [67,68]. In pulmonary rehabilitation, anxiety, depression, dyspnea burden, and poorer baseline status have been associated with non-completion in several studies, although predictors vary across populations [44,74]. Social support may be relevant because it can provide emotional reassurance, practical assistance, symptom interpretation, accountability, and a sense of belonging [37,64,72]. This proposition is consistent with stress-buffering theory, which suggests that social resources may become especially important when people face high demands or emotional strain [37,64,72].

In COPD, caregiver presence and social support have been associated with participation and self-care behaviors, suggesting that the social environment can shape rehabilitation behavior directly [37,38]. Qualitative work shows that pulmonary rehabilitation participants often value peer fellowship, shared illness understanding, group belonging, and encouragement from others who face similar limitations [64,65]. Family-oriented pulmonary rehabilitation research also indicates that caregiver-aware models can support the wider coping system around the patient [66]. In cardiac rehabilitation, social support, expectations, professional encouragement, and perceived fit with daily life are repeatedly identified as influences on attendance and completion [10]. These findings support the hypothesis that retention is socially embedded, especially for patients whose distress would otherwise reduce their capacity to persist [67,68].

#### 6.4.2. Pathways to Staged Retention

The first pathway links support to uptake through encouragement, referral reinforcement, help with transport, and shared decision-making within households [37]. The second pathway links peer belonging to attendance because group connection can transform rehabilitation from an isolated medical obligation into a meaningful social routine [64,65]. The third pathway links support to completion through distress buffering because patients with anxiety or depressive symptoms may continue more reliably when caregivers or peers help regulate discouragement, uncertainty, and fear [38,66]. The fourth pathway links social support to maintenance because family members and peers can reinforce home exercise, lifestyle changes, and symptom-management habits after formal sessions end [44,74].

This hypothesis can be tested with measures such as the Medical Outcomes Study Social Support Survey, caregiver presence, household support indicators, peer-contact frequency, group cohesion measures, and validated depression and anxiety scales [37]. The most direct statistical test would examine support by distress interactions on attendance, completion, and time to dropout. Intervention studies could add caregiver-inclusive orientation, peer mentoring, structured group-belonging practices, or targeted support pathways for patients who screen as high distress and low support. Clinically, this hypothesis suggests that social support should be treated as a modifiable rehabilitation input rather than as background information recorded only at baseline [6,37,44,75].

## 7. Discussion

The central contribution of this review is conceptual rather than confirmatory [39,40]. It adapts established adherence and implementation distinctions to a six-phase rehabilitation sequence and separates observable retention from the provisional processes grouped under rehabilitation resilience [14,15]. This organization may make stage-specific hypotheses more testable, but it has not been shown to outperform existing classifications or to predict outcomes [14,15,54].

Evidence directness remains uneven across conditions and mechanisms [6,9,22,23,24,36]. Cardiac and pulmonary rehabilitation provide the clearest evidence about referral, attendance, and completion, although predictors vary across settings and populations [6,8,9,10,43,44]. Psychological flexibility evidence is concentrated in chronic pain, while alliance evidence is concentrated in musculoskeletal physiotherapy, so the proposed cross-condition bridges remain hypotheses [24,25,51,52,53,58,59,60,61]. Differences in symptom meaning, red flags, clinical severity, and service organization further limit generalization [19,25,26,27].

The framework also changes the interpretation of dropout by separating avoidable disengagement from appropriate discontinuation [20,21]. Early goal attainment, inadequate benefit, adverse events, symptom worsening, changing clinical need, excessive treatment burden, or a shared decision to use another intervention may justify leaving before a standard endpoint [1,20,21]. Retention is therefore desirable only while care remains safe, appropriate, beneficial, and consistent with informed patient goals [1,15,20,21]. Structural barriers also require direct service and policy responses, and mechanism-focused interventions cannot substitute for accessible referral, capacity, affordability, transport, scheduling, and language support [6,9,10,18,19,20].

Empirical testing should begin with longitudinal cohorts that establish temporal ordering among determinants, mechanisms, and stage-specific outcomes [54]. Because flexibility, coherence, self-efficacy, motivation, alliance, and support may influence one another, prespecified competing models are preferable to entering every construct as a parallel predictor [46,47,48,49,50,51,52,53,54]. Intervention trials should follow evidence that a proposed mechanism predicts or mediates a relevant retention phase within the target population [54,61,70]. Administrative process measures should also remain separate from clinical response, adverse events, burden, and goal attainment so that higher retention is not mistaken for better care [15,20,21].

### 7.1. Strengths and Limitations

A strength of this focused review is the integration of rehabilitation participation research with psychological, relational, and multilevel theory in a form that generates falsifiable questions [19,39,40,54]. The six-phase terminology, evidence-status table, and distinction between observable outcomes and provisional mechanisms provide a clearer basis for stage-specific measurement and comparison [14,15,54].

This review also has important methodological limitations [39,40]. Literature identification was purposive and non-systematic, was not independently duplicated, and did not use a prospectively fixed search string or screening denominator, which creates risks of selection and confirmation bias [39,40]. Only English-language sources were used, and no formal study-level risk-of-bias assessment or certainty grading was performed [39,40]. The 76 cited sources include heterogeneous designs, outcomes, and rehabilitation contexts and should not be interpreted as a complete evidence inventory [6,9,12,39].

Many proposed pathways depend on observational, qualitative, or adjacent evidence, and direct mediation evidence for stage-specific retention is limited [10,44,52,61,70]. Generalizability is constrained by differences among cardiac, pulmonary, musculoskeletal, pain, neurological, and multimorbidity rehabilitation and by cultural and health-system variation [6,9,19,24,25,26,27]. The component constructs overlap, rehabilitation resilience has no validated operational measure, and a psychologically oriented synthesis may still underrepresent structural disengagement [14,18,19,54,55]. Patients and the public were not involved in constructing the framework, which may have limited attention to patient-defined goals, burden, adverse experiences, and appropriate discontinuation [20,21]. The model and hypotheses therefore require independent empirical validation before clinical or performance-management use [39,54].

### 7.2. Future Directions

The first priority for future research is to move from cross-sectional prediction toward longitudinal mechanism testing. Baseline-only studies are insufficient because psychological flexibility, self-efficacy, alliance, motivation, and support can change during rehabilitation, especially during the early sessions when dropout risk may emerge [11,59,61]. Future cohorts should measure key mechanisms at intake, after the first few sessions, mid-program, discharge, and follow-up, then model how changes in those mechanisms predict uptake, attendance dose, completion, and maintenance [31,53]. Large registry studies should add psychosocial and relational measures where feasible because clinical and demographic variables alone are unlikely to explain the full pattern of attrition [6,8].

The second priority is to sequence mechanism validation before intervention testing [39,54]. If prospective associations are confirmed within a target population, pragmatic trials could examine brief acceptance-informed onboarding for high-avoidance patients, coherence-building consultations for patients with low illness understanding, autonomy-supportive communication training for rehabilitation staff, or peer and caregiver pathways for patients with high distress or low support [9,24,47,61,64]. Cluster randomized designs may be appropriate for team-level communication and motivational-climate interventions, while safety, burden, benefit, and equity should be monitored alongside retention [15,20,21,27,38]. Hybrid and digital rehabilitation models should examine whether remote formats preserve alliance, autonomy support, competence feedback, belonging, and access rather than reporting completion alone [75,76].

The third priority is measurement standardization [11,12,13,15]. Studies should report referral and eligibility, uptake or initiation, early attendance, ongoing attendance or dose, completion or planned discharge, and post-program maintenance, with time to dropout treated as a longitudinal metric [7,11,12,13,14,15]. Prescribed and attended sessions, service and patient cancellations, individualized dose changes, reasons for discontinuation, and follow-up timing should be explicit [12,13,23,41]. Psychological measures should be selected according to one prespecified mechanism and interpreted with clinical severity and structural access variables [30,34,53,54].

The fourth priority is equity. Retention mechanisms do not operate in a social vacuum because rurality, transport, socioeconomic status, language, health literacy, comorbidity, caregiving responsibilities, and digital exclusion can all shape whether psychological and relational resources can be translated into attendance [6,8,9]. Future models should therefore avoid becoming psychologically rich but structurally blind. The strongest future framework will be multilevel, combining person-centered adaptation theory with service design, access, caregiver context, and social determinants of health [1,4,37].

## 8. Conclusions

On the basis of this non-systematic conceptual synthesis, rehabilitation retention may be more informative when reported across six phases than as a single completion endpoint [7,11,12,13,14,15]. Rehabilitation resilience is offered as a provisional multilevel heuristic that separates observable participation from hypothesized patient, relational, and system processes [4,19,55]. The four author-generated hypotheses draw on uneven direct and indirect evidence and require prospective, condition-specific testing with explicit clinical safety and structural determinants [6,9,24,27,36,54,61]. Clinical services should seek to reduce avoidable disengagement while respecting planned discontinuation, changing clinical needs, treatment burden, and informed patient goals [1,20,21].

## Figures and Tables

**Table 1:** Proposed operational framework for six-phase rehabilitation retention and measurement [7,11,12,13,14,15,23,41].

Retention Phase	Working Definition	Main Behavioral Question	Likely Dominant Mechanisms	Example Indicators and Denominator
Referral and eligibility	Identifying an eligible patient and offering an appropriate program	Does the system provide an appropriate opportunity to participate?	Eligibility rules, referral practice, capacity, waiting time, communication, equity	Eligible patients referred; referral method; waiting time; denominator: clinically eligible patients
Uptake or initiation	Accepting referral and attending assessment or the first rehabilitation session	Does the patient begin rehabilitation?	Illness coherence, perceived need, referral endorsement, access, uncertainty, self-efficacy	Referral acceptance and first-session attendance; denominator: referred patients
Early attendance	Participation during a prespecified opening period	Does the patient return after initial contact?	Initial tolerability, safety, symptom interpretation, self-efficacy, early alliance, access	Attended, patient-missed, and service-cancelled sessions during the first 2–4 offered sessions
Ongoing attendance or dose	Participation across the individualized active program	What proportion of prescribed care is received over time?	Clinical response, symptoms, alliance, motivation, transport, treatment burden, support	Attendance proportion, dose changes, and time to dropout; denominator: individualized prescription adjusted for service cancellations
Completion or planned discharge	Reaching protocol dose, agreed goals, or a planned discharge decision	Is care completed or discontinued for an appropriate, documented reason?	Clinical benefit, adverse events, goals, burden, shared decision-making, alliance	Protocol completion, goal attainment, and planned or unplanned discontinuation with reason
Post-program maintenance	Sustained exercise or self-management after supervised care	Does behavior continue after supervised care ends?	Autonomous motivation, self-efficacy, habit formation, social support, values-based action	Behavior or activity at prespecified follow-up, preferably 3, 6, and 12 months

**Table 2:** Author-proposed adaptation pathways linking patient experience, intermediate mechanisms, and hypothesized retention associations [4,6,54].

Pathway	Immediate Process	Intermediate Mechanism	Hypothesized Retention Association
Symptom discomfort to flexible action	Patient notices discomfort without automatic avoidance	Reduced fear, reduced experiential avoidance, values-based persistence	Potentially greater attendance and completion
Low coherence to clarified rationale	Patient understands why rehabilitation matters	Lower uncertainty, greater perceived control, stronger perceived benefit	Potentially greater uptake and early attendance
Low confidence to mastery	Patient experiences graded success and feedback	Higher self-efficacy, lower helplessness, stronger task engagement	Potentially greater attendance and completion
Controlling care to autonomy support	Clinician uses noncontrolling language and shared goals	Greater autonomous motivation and competence	Potentially greater completion and maintenance
Weak relationship to alliance	Patient feels understood and collaborates on barriers	More disclosure, better tailoring, lower silent disengagement	Potentially greater attendance and completion
Isolation to belonging	Patient receives caregiver, peer, or group support	Emotional buffering, accountability, normalization	Potentially greater retention, particularly under distress

**Table 3:** Author-proposed mechanistic hypotheses, measures, study designs, and candidate clinical implications for rehabilitation retention [39,54].

Author-Proposed Hypothesis	Core Mechanism	Example Measures	Most Relevant Retention Phases	Suggested Study Design	Clinical Implication
H1. Psychological flexibility and acceptance	Reduced experiential avoidance, values-based action under discomfort, flexible response to symptoms	CPAQ, CPAQ-8, PIPS, broader psychological flexibility measures, symptom-avoidance indicators	Early and ongoing attendance; completion; maintenance	Prospective cohort, pragmatic ACT-informed trial, mediation analysis	Screen avoidance with severity and safety
H2. Illness coherence, uncertainty, and self-efficacy	Better sense-making, lower uncertainty, greater perceived control, confidence in rehabilitation tasks	Brief IPQ, IPQ-R, SEMCD, PRAISE, uncertainty measures	Initiation; early attendance; dose; maintenance	Longitudinal cohort, intake intervention, mixed-methods study	Tailored orientation, expectation-setting, graded mastery
H3. Autonomy support and alliance	Need satisfaction, autonomous motivation, collaborative problem-solving, stronger disclosure of barriers	HCCQ, WAI rehabilitation versions, need-satisfaction scales, motivation scales	Attendance; completion or planned discharge; maintenance	Cluster RCT; multilevel or reciprocal analysis	Shared goals and treatment adaptation
H4. Social support and belonging	Distress buffering, practical help, accountability, normalization, belonging	MOS-SSS, caregiver presence, peer-contact frequency, group cohesion, HADS or similar mood scales	Uptake; attendance; planned discharge; maintenance	Moderation analysis, caregiver-inclusive trial, peer-support intervention	Preference-matched support; monitor harms

**Table 4:** Evidence-status map distinguishing direct rehabilitation evidence, adjacent evidence, and author-proposed relationships [6,9,22,23,24,36,39,54].

Proposition	Direct Rehabilitation Evidence	Adjacent or Indirect Evidence	Evidence Status and Uncertainty
H1. Psychological flexibility and acceptance	Chronic pain rehabilitation associations and mediation analyses [24,25,58,59,60,61,70]	Limited direct evidence for staged retention in cardiac, pulmonary, neurological, or multimorbidity rehabilitation [6,9,26]	The cross-condition retention pathway is author-generated and unvalidated
H2. Illness coherence and self-efficacy	Cardiac attendance and pulmonary rehabilitation self-efficacy or behavior [31,32,34,71]	General chronic-disease evidence on uncertainty, illness perceptions, and self-efficacy [28,30,62,63]	Associations have direct support in selected settings; mediation and stage specificity remain uncertain
H3. Autonomy support and alliance	Cardiac exercise motivation and musculoskeletal alliance evidence [47,48,51,52,53]	Self-determination theory and health-context evidence [35,46,49,50]	Causal retention effects and reciprocal ordering remain untested
H4. Social support and peer belonging	Pulmonary and cardiac participation, self-care, and qualitative evidence [18,37,38,64,66,67,68]	Stress-buffering theory and chronic-illness family evidence [69,72]	Moderation, optimal support type, and possible negative effects remain uncertain
Clinical and structural determinants	Direct evidence for severity, referral, transport, cost, and program factors [6,8,9,10,18,43,44,45]	Ecological and treatment-burden models [19,20]	These determinants may act directly and do not require psychological mediation

## Data Availability

No new data were created or analyzed in this study. Data sharing is not applicable to this article.

## Abbreviations

The following abbreviations are used in this manuscript:

ACT	Acceptance and Commitment Therapy
COPD	Chronic Obstructive Pulmonary Disease
CPAQ	Chronic Pain Acceptance Questionnaire
CPAQ-8	8-item Chronic Pain Acceptance Questionnaire
HADS	Hospital Anxiety and Depression Scale
HCCQ	Health Care Climate Questionnaire
IPQ	Illness Perception Questionnaire
IPQ-R	Revised Illness Perception Questionnaire
MOS-SSS	Medical Outcomes Study Social Support Survey
PIPS	Psychological Inflexibility in Pain Scale
PRAISE	Pulmonary Rehabilitation Adapted Index of Self-Efficacy
RCT	Randomized Controlled Trial
SEMCD	Self-Efficacy for Managing Chronic Disease
WAI	Working Alliance Inventory

## References

[1] World Health Organization (2024). Rehabilitation. https://www.who.int/news-room/fact-sheets/detail/rehabilitation.

[2] Cieza A., Causey K., Kamenov K., Hanson S.W., Chatterji S., Vos T. (2020). Global estimates of the need for rehabilitation based on the Global Burden of Disease study 2019: A systematic analysis for the Global Burden of Disease Study 2019. Lancet.

[3] Stucki G., Bickenbach J., Gutenbrunner C., Melvin J. (2018). Rehabilitation: The health strategy of the 21st century. J. Rehabil. Med..

[4] Carroll S., Moon Z., Hudson J., Hulme K., Moss-Morris R. (2022). An Evidence-Based Theory of Psychological Adjustment to Long-Term Physical Health Conditions: Applications in Clinical Practice. Biopsychosoc. Sci. Med..

[5] Jansons P.S., Haines T.P., O’Brien L. (2017). Interventions to achieve ongoing exercise adherence for adults with chronic health conditions who have completed a supervised exercise program: Systematic review and meta-analysis. Clin. Rehabil..

[6] Resurrección D.M., Moreno-Peral P., Gómez-Herranz M., Rubio-Valera M., Pastor L., Caldas de Almeida J.M., Motrico E. (2019). Factors associated with non-participation in and dropout from cardiac rehabilitation programmes: A systematic review of prospective cohort studies. Eur. J. Cardiovasc. Nurs..

[7] Jones A.W., Taylor A., Gowler H., O’Kelly N., Ghosh S., Bridle C. (2017). Systematic review of interventions to improve patient uptake and completion of pulmonary rehabilitation in COPD. ERJ Open Res..

[8] Thomas E.E., Le Grande M., Phillips S., Cartledge S., Poulter R., Murphy B.M. (2025). Predictors of Cardiac Rehabilitation Attendance and Completion: Analysis of 33,055 Patients from the Queensland Cardiac Outcomes Registry (2020–2022). Heart Lung Circ..

[9] Cox N.S., Oliveira C.C., Lahham A., Holland A.E. (2017). Pulmonary rehabilitation referral and participation are commonly influenced by environment, knowledge, and beliefs about consequences: A systematic review using the Theoretical Domains Framework. J. Physiother..

[10] Clark A.M., King-Shier K.M., Thompson D.R., Spaling M.A., Duncan A.S., Stone J.A., Jaglal S.B., Angus J.E. (2012). A qualitative systematic review of influences on attendance at cardiac rehabilitation programs after referral. Am. Heart J..

[11] Ley C., Putz P. (2024). Efficacy of interventions and techniques on adherence to physiotherapy in adults: An overview of systematic reviews and panoramic meta-analysis. Syst. Rev..

[12] Kenny M., Ranabahu T., Vallance P., Zhang Y., Gurr J., Färnqvist K., Rathi S., Cridland K., Munteanu S.E., Malliaras P. (2023). Exercise adherence in trials of therapeutic exercise interventions for common musculoskeletal conditions: A scoping review. Musculoskelet. Sci. Pract..

[13] Hawley-Hague H., Horne M., Skelton D.A., Todd C. (2016). Review of how we should define (and measure) adherence in studies examining older adults’ participation in exercise classes. BMJ Open.

[14] Vrijens B., De Geest S., Hughes D.A., Kardas P., Demonceau J., Ruppar T., Dobbels F., Fargher E., Morrison V., Lewek P. (2012). A new taxonomy for describing and defining adherence to medications. Br. J. Clin. Pharmacol..

[15] Proctor E., Silmere H., Raghavan R., Hovmand P., Aarons G., Bunger A., Griffey R., Hensley M. (2011). Outcomes for implementation research: Conceptual distinctions, measurement challenges, and research agenda. Adm. Policy Ment. Health Ment. Health Serv. Res..

[16] Babatunde F., MacDermid J., MacIntyre N. (2017). Characteristics of therapeutic alliance in musculoskeletal physiotherapy and occupational therapy practice: A scoping review of the literature. BMC Health Serv. Res..

[17] Burke C.A., Seidler K.J., Rethorn Z.D., Hoenig H., Allen K., Tabriz A.A., Norman K., Murphy-McMillan L.K., Sharpe J., Joseph L.M. (2024). Interventions to Improve Long-Term Adherence to Physical Rehabilitation: A Systematic Review. J. Geriatr. Phys. Ther..

[18] Oates G.R., Hamby B.W., Stepanikova I., Knight S.J., Bhatt S.P., Hitchcock J., Schumann C., Dransfield M.T. (2017). Social Determinants of Adherence to Pulmonary Rehabilitation for Chronic Obstructive Pulmonary Disease. COPD J. Chronic Obstr. Pulm. Dis..

[19] Richard L., Gauvin L., Raine K. (2011). Ecological models revisited: Their uses and evolution in health promotion over two decades. Annu. Rev. Public Health.

[20] Mair F.S., May C.R. (2014). Thinking about the burden of treatment. BMJ.

[21] Elwyn G., Frosch D.L., Thomson R., Joseph-Williams N., Lloyd A., Kinnersley P., Cording E., Tomson D., Dodd C., Rollnick S. (2012). Shared decision making: A model for clinical practice. J. Gen. Intern. Med..

[22] Essery R., Geraghty A.W.A., Kirby S., Yardley L. (2017). Predictors of adherence to home-based physical therapies: A systematic review. Disabil. Rehabil..

[23] Mahmood A., Nayak P., Deshmukh A., English C., N M., Solomon M J., B U. (2023). Measurement, determinants, barriers, and interventions for exercise adherence: A scoping review. J. Bodyw. Mov. Ther..

[24] Hughes L.S., Clark J., Colclough J.A., Dale E., McMillan D. (2017). Acceptance and Commitment Therapy (ACT) for Chronic Pain: A Systematic Review and Meta-Analyses. Clin. J. Pain.

[25] Leeuw M., Goossens M.E.J.B., Linton S.J., Crombez G., Boersma K., Vlaeyen J.W.S. (2007). The Fear-Avoidance Model of Musculoskeletal Pain: Current State of Scientific Evidence. J. Behav. Med..

[26] Barker K., Holland A.E., Skinner E.H., Lee A.L. (2023). Clinical Outcomes Following Exercise Rehabilitation in People with Multimorbidity: A Systematic Review. J. Rehabil. Med..

[27] Spruit M.A., Singh S.J., Garvey C., ZuWallack R., Nici L., Rochester C., Hill K., Holland A.E., Lareau S.C., Man W.D.-C. (2013). An official American Thoracic Society/European Respiratory Society statement: Key concepts and advances in pulmonary rehabilitation. Am. J. Respir. Crit. Care Med..

[28] Gibson B., Rosser B.A., Schneider J., Forshaw M.J. (2023). The role of uncertainty intolerance in adjusting to long-term physical health conditions: A systematic review. PLoS ONE.

[29] Hayes S.C., Luoma J.B., Bond F.W., Masuda A., Lillis J. (2006). Acceptance and Commitment Therapy: Model, processes and outcomes. Behav. Res. Ther..

[30] Broadbent E., Petrie K.J., Main J., Weinman J. (2006). The Brief Illness Perception Questionnaire. J. Psychosom. Res..

[31] Liacos A., McDonald C.F., Mahal A., Hill C.J., Lee A.L., Burge A.T., Moore R., Nicolson C., O’Halloran P., Cox N.S. (2019). The Pulmonary Rehabilitation Adapted Index of Self-Efficacy (PRAISE) tool predicts reduction in sedentary time following pulmonary rehabilitation in people with chronic obstructive pulmonary disease (COPD). Physiotherapy.

[32] Blair J., Angus N.J., Lauder W.J., Atherton I., Evans J., Leslie S.J. (2014). The influence of non-modifiable illness perceptions on attendance at cardiac rehabilitation. Eur. J. Cardiovasc. Nurs..

[33] Gentili C., Rickardsson J., Zetterqvist V., Simons L.E., Lekander M., Wicksell R.K. (2019). Psychological Flexibility as a Resilience Factor in Individuals with Chronic Pain. Front. Psychol..

[34] Vincent E., Sewell L., Wagg K., Deacon S., Williams J., Singh S. (2011). Measuring a Change in Self-Efficacy Following Pulmonary Rehabilitation: An Evaluation of the PRAISE Tool. Chest.

[35] Teixeira P.J., Carraça E.V., Markland D., Silva M.N., Ryan R.M. (2012). Exercise, physical activity, and self-determination theory: A systematic review. Int. J. Behav. Nutr. Phys. Act..

[36] Hall A.M., Ferreira P.H., Maher C.G., Latimer J., Ferreira M.L. (2010). The Influence of the Therapist-Patient Relationship on Treatment Outcome in Physical Rehabilitation: A Systematic Review. Phys. Ther..

[37] Chen Z., Fan V.S., Belza B., Pike K., Nguyen H.Q. (2017). Association between Social Support and Self-Care Behaviors in Adults with Chronic Obstructive Pulmonary Disease. Ann. Am. Thorac. Soc..

[38] Figueiredo D., Cruz J., Jácome C., Marques A. (2016). Exploring the Benefits to Caregivers of a Family-Oriented Pulmonary Rehabilitation Program. Respir. Care.

[39] Baethge C., Goldbeck-Wood S., Mertens S. (2019). SANRA-a scale for the quality assessment of narrative review articles. Res. Integr. Peer Rev..

[40] Green B.N., Johnson C.D., Adams A. (2006). Writing narrative literature reviews for peer-reviewed journals: Secrets of the trade. J. Chiropr. Med..

[41] Bollen J.C., Dean S.G., Siegert R.J., Howe T.E., Goodwin V.A. (2014). A systematic review of measures of self-reported adherence to unsupervised home-based rehabilitation exercise programmes, and their psychometric properties. BMJ Open.

[42] Gunnes M., Langhammer B., Aamot I.-L., Lydersen S., Ihle-Hansen H., Indredavik B., Reneflot K.H., Schroeter W., Askim T., LAST Collaboration Group (2019). Adherence to a Long-Term Physical Activity and Exercise Program After Stroke Applied in a Randomized Controlled Trial. Phys. Ther..

[43] Brown A.T., Hitchcock J., Schumann C., Wells J.M., Dransfield M.T., Bhatt S.P. (2016). Determinants of successful completion of pulmonary rehabilitation in COPD. Int. J. Chronic Obstr. Pulm. Dis..

[44] Yohannes A.M., Casaburi R., Dryden S., Hanania N.A. (2022). Predictors of premature discontinuation and prevalence of dropouts from a pulmonary rehabilitation program in patients with chronic obstructive pulmonary disease. Respir. Med..

[45] Hayton C., Clark A., Olive S., Browne P., Galey P., Knights E., Staunton L., Jones A., Coombes E., Wilson A.M. (2013). Barriers to pulmonary rehabilitation: Characteristics that predict patient attendance and adherence. Respir. Med..

[46] Deci E.L., Ryan R.M. (2008). Self-determination theory: A macrotheory of human motivation, development, and health. Can. Psychol./Psychol. Can..

[47] Russell K.L., Bray S.R. (2010). Promoting self-determined motivation for exercise in cardiac rehabilitation: The role of autonomy support. Rehabil. Psychol..

[48] Sweet S.N., Fortier M.S., Strachan S.M., Blanchard C.M., Boulay P. (2014). Testing a Longitudinal Integrated Self-Efficacy and Self-Determination Theory Model for Physical Activity Post-Cardiac Rehabilitation. Health Psychol. Res..

[49] Ng J.Y.Y., Ntoumanis N., Thøgersen-Ntoumani C., Deci E.L., Ryan R.M., Duda J.L., Williams G.C. (2012). Self-Determination Theory Applied to Health Contexts: A Meta-Analysis. Perspect. Psychol. Sci..

[50] Czajkowska Z., Wang H., Hall N.C., Sewitch M., Körner A. (2017). Validation of the English and French versions of the Brief Health Care Climate Questionnaire. Health Psychol. Open.

[51] Kinney M., Seider J., Beaty A.F., Coughlin K., Dyal M., Clewley D. (2020). The impact of therapeutic alliance in physical therapy for chronic musculoskeletal pain: A systematic review of the literature. Physiother. Theory Pract..

[52] Moore A.J., Holden M.A., Foster N.E., Jinks C. (2020). Therapeutic alliance facilitates adherence to physiotherapy-led exercise and physical activity for older adults with knee pain: A longitudinal qualitative study. J. Physiother..

[53] Paap M.D., Schrier M.S.E., Dijkstra P.P.U. (2019). Development and validation of the Working Alliance Inventory Dutch version for use in rehabilitation setting. Physiother. Theory Pract..

[54] VanderWeele T.J. (2016). Mediation analysis: A practitioner’s guide. Annu. Rev. Public Health.

[55] Gheshlagh R.G., Sayehmiri K., Ebadi A., Dalvandi A., Dalvand S., Maddah S.B., Tabrizi K.N. (2017). The relationship between mental health and resilience: A systematic review and meta-analysis. Iran. Red Crescent Med. J..

[56] Rovner G.S., Arestedt K., Gerdle B., Börsbo B., McCracken L.M. (2014). Psychometric properties of the 8-item Chronic Pain Acceptance Questionnaire (CPAQ-8) in a Swedish chronic pain cohort. J. Rehabil. Med..

[57] Freund T., Gensichen J., Goetz K., Szecsenyi J., Mahler C. (2013). Evaluating self-efficacy for managing chronic disease: Psychometric properties of the six-item Self-Efficacy Scale in Germany. J. Eval. Clin. Pract..

[58] Vowles K.E., Sowden G., Ashworth J. (2014). A Comprehensive Examination of the Model Underlying Acceptance and Commitment Therapy for Chronic Pain. Behav. Ther..

[59] Scott W., Hann K.E.J., McCracken L.M. (2016). A Comprehensive Examination of Changes in Psychological Flexibility Following Acceptance and Commitment Therapy for Chronic Pain. J. Contemp. Psychother..

[60] McCracken L.M. (2024). Psychological Flexibility, Chronic Pain, and Health. Annu. Rev. Psychol..

[61] Murillo C., Vo T.-T., Vansteelandt S., Harrison L.E., Cagnie B., Coppieters I., Chys M., Timmers I., Meeus M. (2022). How do psychologically based interventions for chronic musculoskeletal pain work? A systematic review and meta-analysis of specific moderators and mediators of treatment. Clin. Psychol. Rev..

[62] Lorig K.R., Sobel D.S., Ritter P.L., Laurent D., Hobbs M. (2001). Effect of a self-management program on patients with chronic disease. Eff. Clin. Pract. ECP.

[63] Skojec T.A., Davidson T.M., Kelechi T.J. (2025). The relationship between uncertainty in illness and psychological adjustment to chronic illness. J. Health Psychol..

[64] Halding A.-G., Wahl A., Heggdal K. (2010). ‘Belonging’. ‘Patients’ experiences of social relationships during pulmonary rehabilitation. Disabil. Rehabil..

[65] Guo S.-E., Bruce A. (2014). Improving Understanding of and Adherence to Pulmonary Rehabilitation in Patients with COPD: A Qualitative Inquiry of Patient and Health Professional Perspectives. PLoS ONE.

[66] Marques A. (2024). Pulmonary rehabilitation and family/friend caregivers: The hidden reciprocal relationship improving outcomes in chronic respiratory diseases. Expert Rev. Respir. Med..

[67] McGrady A., McGinnis R., Badenhop D., Bentle M., Rajput M. (2009). Effects of Depression and Anxiety on Adherence to Cardiac Rehabilitation. J. Cardiopulm. Rehabil. Prev..

[68] Fan Y., Shen B.-J., Ho M.-H.R. (2024). Loneliness, perceived social support, and their changes predict medical adherence over 12 months among patients with coronary heart disease. Br. J. Health Psychol..

[69] Rosland A.-M., Heisler M., Piette J.D. (2012). The impact of family behaviors and communication patterns on chronic illness outcomes: A systematic review. J. Behav. Med..

[70] Vowles K.E., Witkiewitz K., Sowden G., Ashworth J. (2014). Acceptance and Commitment Therapy for Chronic Pain: Evidence of Mediation and Clinically Significant Change Following an Abbreviated Interdisciplinary Program of Rehabilitation. J. Pain.

[71] French D.P., Cooper A., Weinman J. (2006). Illness perceptions predict attendance at cardiac rehabilitation following acute myocardial infarction: A systematic review with meta-analysis. J. Psychosom. Res..

[72] Uchino B.N. (2006). Social Support and Health: A Review of Physiological Processes Potentially Underlying Links to Disease Outcomes. J. Behav. Med..

[73] McCracken L.M., Vowles K.E. (2014). Acceptance and commitment therapy and mindfulness for chronic pain: Model, process, and progress. Am. Psychol..

[74] Boutou A.K., Tanner R.J., Lord V.M., Hogg L., Nolan J., Jefford H., Corner E.J., Falzon C., Lee C., Garrod R. (2014). An evaluation of factors associated with completion and benefit from pulmonary rehabilitation in COPD. BMJ Open Respir. Res..

[75] Candy S., Reeve J., Dobson R., Whittaker R., Garrett J., Warren J., Calder A., Tane T., Robertson T., Rashid U. (2023). The Impact of Patient Preference on Attendance and Completion Rates at Centre-Based and mHealth Pulmonary Rehabilitation: A Non-Inferiority Pragmatic Clinical Trial. Int. J. Chronic Obstr. Pulm. Dis..

[76] Rodríguez-Romero R., Falces C., Kostov B., García-Planas N., Blat-Guimerà E., Alvira-Balada M.C., López-Poyato M., Benito-Serrano M.L., Vidiella-Piñol I., Zamora-Sánchez J.J. (2022). A motivational interview program for cardiac rehabilitation after acute myocardial infarction: Study protocol of a randomized controlled trial in primary healthcare. BMC Prim. Care.

